# In Vitro Antidiabetic, Antioxidant Activity, and Probiotic Activities of *Lactiplantibacillus plantarum* and *Lacticaseibacillus paracasei* Strains

**DOI:** 10.1007/s00284-021-02588-5

**Published:** 2021-07-02

**Authors:** GaYeong Won, Soo-Im Choi, NaYeong Park, Ji-Eun Kim, Chang-Ho Kang, Gun-Hee Kim

**Affiliations:** 1 Department of Health Functional New Materials, Duksung Women’s University, Seoul, 01369 Korea; 2 Department of Food and Nutrition, Duksung Women’s University, Seoul, 01369 Korea; 3 MEDIOGEN, Co., Ltd., Jecheon, 27159 Korea

## Abstract

Diabetes, a chronic metabolic disorder, is characterized by persistent hyperglycemia. This study aimed to evaluate the hypoglycemic and antioxidant activities of lactic acid bacteria strains isolated from humans and food products and investigate the probiotic properties of the selected four strains. The hypoglycemic activity of the isolated strains was examined by evaluating the α-glucosidase and α-amylase inhibitory activities. The antioxidant activity was measured using the DPPH, ABTS, and FRAP assays. Four strains (*Lactiplantibacillus plantarum* MG4229, MG4296, MG5025, and *Lacticaseibacillus paracasei* MG5012) exhibited potent α-glucosidase inhibitory (>75%) and α-amylase inhibitory (>85%) activities, which were comparable to those of acarbose (>50%; 1000 μg/mL). Similarly, the radical scavenging and antioxidant activities of the four strains were comparable to those of ascorbic acid (50 μg/mL). Additionally, the probiotic properties of the four selected strains were examined based on acid and bile salt tolerance, auto-aggregation ability, and antibiotic resistance. The four strains were resistant to pH 2 (>50% of survivability) and 0.5% bile salt (>80% of survivability). Therefore, we suggest that the selected strains with hypoglycemic, antioxidant, probiotic properties can potentially prevent diabetes.

**Supplementary Information:**

The online version contains supplementary material available at 10.1007/s00284-021-02588-5.

## Introduction

The World Health Organization (WHO) has defined probiotics as live microorganisms that provide health benefits to the host when administered in appropriate amounts. Lactic acid bacteria (LAB), which are generally regarded as safe (GRAS) bacteria, are widely used to develop products with functional and probiotic properties because of their resistance to low pH and bile salts in the intestine [1]. Probiotics are reported to alleviate lactose intolerance, diarrhea, or peptic ulcers and exhibit anti-allergic, antifungal, anticancer, and immunomodulatory properties [2]. Short-chain fatty acids (SCFAs), such as acetate, propionate, and butyrate, are produced during the intestinal microbial metabolization of carbohydrates. Additionally, SCFAs can be an energy source for the intestinal epithelial cells, strengthen the immune system, mitigate inflammation, and regulate metabolism [3]. The demand for probiotics is rapidly increasing owing to the enhanced consumer awareness of gut health and the beneficial effects of probiotics.

Diabetes mellitus (DM) is a chronic metabolic disease characterized by persistent hyperglycemia caused due to decreased insulin production or impaired insulin activity. In patients with diabetes, glucose homeostasis is dysregulated due to impaired insulin secretion and activity. Type 2 diabetes mellitus (T2DM), the most common type of insulin resistance, is caused by genetic factors, obesity, westernized eating habits, and lack of exercise [4]. T2DM is usually treated with drugs, but modulation of gut microbial composition using probiotics could be an essential factor in neutralizing metabolic diseases, including obesity and diabetes [5]. Recent studies have reported that probiotics improve the symptoms of diabetes by regulating the intestinal microbiota composition, increasing insulin sensitivity, and mitigating autoimmune responses [6]. Some studies have also demonstrated that beneficial gut bacteria decrease blood glucose levels by regulating the release of enzymes and hormones [7].

Glucosidase enzymes are expressed at the intestinal brush border and degrade complex oligosaccharides and disaccharides into glucose, which is subsequently absorbed in the intestine [8]. The concentration and activity of intestinal glucosidases determine the postprandial blood glucose levels [9, 10]. Inhibition of α-glucosidase, which catalyzes the final stage of the process of polysaccharides digestion, decreases postprandial glucose levels by delaying the release and absorption of glucose and consequently inhibits postprandial hyperglycemia, delays carbohydrate metabolism, and prevents excessive glucose absorption. Therefore, the inhibition of carbohydrate hydrolases, such as α-glucosidase, is an effective strategy for mitigating postprandial hyperglycemia in T2DM [11]. Acarbose, voglibose, and miglitol, which are commercially available α-glucosidase targeting inhibitors, competitively bind to enzymes and inhibit their activity. However, these inhibitors are associated with the development of gastrointestinal complications, such as abdominal distension and diarrhea, which has limited their clinical application [12].

In our preliminary study, the α-glucosidase inhibitory activities of 253 LAB strains isolated from humans or food products were screened. In total, 17 strains with α-glucosidase inhibitory activity were identified. The findings of this study indicated that four strains (*Lactiplantibacillus plantarum* and *Lacticaseibacillus paracasei*) with potential α-glucosidase inhibitory, α-amylase inhibitory, antioxidant, and probiotic activities could be potential novel probiotics to treat diabetes.

## Materials and Methods

### Experimental Materials

The probiotic candidates (235 strains) used in this study were supplied by MEDIOGEN Co., Ltd. (Jecheon, Korea). *Lactobacilli* de Man, Rogosa, and Sharpe (MRS) agar broth, brain heart infusion agar (BHI), tryptic soy agar (TSA), and phosphate-buffered saline (PBS) were purchased from Difco Co. (MI, USA). The API 50 CHL and API ZYM kits used to identify the LAB were purchased from BioMérieux (Marcy-l’Etoile, France). α-Glucosidase, p-nitrophenyl α-d-glucopyranoside (PNPG), α-amylase, 3,5-dinitrosalicylic acid (DNS), 2,2-diphenyl-1-picrylhydrazyl (DPPH), 2,2′-azino-bis (3-ethylbenzothiazoline-6-sulfonic acid) (ABTS), 2,4,6-tripyridyl-S-triazine (TPTZ), iron (III) chloride (FeCl_3_), bile salts (Oxgall), and other reagents were purchased from Sigma-Aldrich Chemical Co. (MO, USA).

### Identification of Strains

The selected strains were identified using 16S rRNA gene sequencing, which was performed at Sol Gent Co. (Daejeon, Korea) using universal rRNA gene primers (27F and 1492R). The 16S rRNA gene sequencing data were retrieved from the EzBioCloud database (http://www.ezbiocloud.net/). A phylogenetic tree was constructed using MEGA-X with the neighbor-joining method [13].

### Evaluation of In Vitro Antidiabetic and Antioxidant Activities

#### Preparation of Culture Supernatant (CS) and Intact Cells (ICs)

The α-glucosidase and α-amylase inhibitory activities of the isolated strains were measured using CS. To prepare CS, each strain was cultured in MRS broth at 37 °C for 15 h, centrifuged at 800 × *g* for 15 min at 4 °C, and the supernatant was filtered through a 0.2-μm syringe filter. To measure the antioxidant activity of the strains, the ICs were prepared following the methods of Lin and Chang [14]. The strains were cultured at 37 °C for 18 h and centrifuged at 7000 × *g* for 20 min at 4 °C. The recovered pellet (ICs) was washed three times with PBS and resuspended in PBS. The ICs samples were stored at – 80 °C until use. In addition, appropriate cell types (washed cell pellets or colonies) were used to assess probiotic properties, including acid and bile salt resistance.

#### α-Glucosidase Inhibitory Activity

α-glucosidase inhibitory activity of the strains was measured according to the methods described by Chen et al. [15]. Briefly, 25 μL of CS was added to a reaction mixture containing 150 μL of 0.01 M PBS (pH 7.0) and 75 μL of 0.02 M PNPG solution, and pre-incubated at 37 °C for 10 min. The reaction was initiated with the addition of 50 μL α-glucosidase (0.17 units/mL) and the sample was incubated at 37 °C for 10 min. Next, the reaction was terminated with the addition of 1 mL of 0.1 M Na_2_CO_3_. The amount of p-nitrophenol released was determined by measuring the absorbance at 405 nm. The inhibition was calculated as follows:

1 $${\text{Inhibition}}\left( \% \right) = \left[ {\frac{{1 - \left( {C - D} \right)}}{{\left( {A - B} \right)}}} \right] \times 100,$$

where *A* is the absorbance with α-glucosidase but without sample, *B* is the absorbance without α-glucosidase and sample, *C* is the absorbance with α-glucosidase and sample, and D is the absorbance without α-glucosidase but with the sample.

#### α-Amylase Inhibitory Activity

α-amylase inhibitory activity of the strains was evaluated as described by Vankudre et al. [16]. Briefly, 250 μL of CS was added to 250 μL of α-amylase solution (0.5 mg/mL) and pre-incubated at 25 °C for 10 min. The reaction mixture was then incubated with 250 μL of starch solution (1% w/v in 0.02 M sodium phosphate buffer) at 25 °C for 10 min. Next, the reaction was terminated with the addition of 500 μL of DNS color reagent (96 mM DNS and 5.31 M sodium potassium tartrate in 2 M sodium hydroxide solution). The reaction mixture was then boiled for 5 min, allowed to cool, and diluted four-fold with water. The absorbance was measured at 540 nm. The inhibition was calculated as follows:

2 $${\text{Inhibition}}\,\left( \% \right)\, = \,\left[ {\frac{{\left( {A\,{-}\,B} \right)}}{A}} \right]\, \times \,100,$$

where *A* is the absorbance of the control and *B* is the absorbance of the sample.

### DPPH Radical Scavenging Assay

The DPPH radical scavenging ability of the strains was determined following the protocols of Lim [17]. Briefly, 100 μL of ICs and 100 μL of 0.4 mM DPPH solution were added to a 96-well plate and reacted in the dark for 30 min at 20 °C. The sample buffer alone in the reaction mixture served as the control. DPPH radical scavenging (%) was measured based on the decrease in absorbance at 540 nm as follows:

3 $${\text{DPPH}}\,{\text{radical}}\,{\text{scavenging}}\,{\text{ability}}\,\left( \% \right)\, = \,\left[ {1 - \left( {\frac{B}{A}} \right)} \right]\, \times \,100,$$

where *A* is the absorbance of the control and *B* is the absorbance of the sample.

### ABTS Radical Scavenging Assay

The ABTS radical scavenging ability of the strains was performed according to Re et al. [18]. Briefly, 7.4 mM ABTS was reacted with 2.6 mM potassium persulfate for 24 h in the dark at room temperature. The solution was diluted with PBS to 0.70 ± 0.03 at 734 nm, immediately before the assay. ICs (20 μL) were added into a 96-well plate containing 180 μL ABTS radical solution and incubated in the dark for 10 min. The ABTS radical scavenging (%) was monitored by measuring absorption at 734 nm, as follows:

4 $${\text{ABTS}}\,{\text{radical}}\,{\text{scavenging}}\,{\text{activity}}\,\left( \% \right)\, = \,\left[ {1 - \left( {\frac{B}{A}} \right)} \right]\, \times \,100,$$

where *A* is the absorbance of the control and *B* is the absorbance of the sample.

### Ferric Reducing Antioxidant Power (FRAP) Assay

FRAP assay of the strains was performed following the method described by Benzie and Strain with modifications [19]. To prepare FRAP reagent, a solution comprising 0.3 M sodium acetate buffer (pH 3.6), 10 mM TPTZ, and 0.02 M FeCl_3_·6H_2_O in the ratio of 10:1:1 (v/v/v) was prepared and incubated for 15 min at 37 °C. ICs (50 μL) were incubated with 150 μL of FRAP reagent for 20 min in a 96-well plate in the dark. The absorbance was measured at 593 nm. FRAP values were converted using the standard curve of the FeSO_4_·7H_2_O solution.

### In Vitro Characterization of Probiotic Properties

#### Acid and Bile Salt Tolerance

The acid and bile salt tolerance of the selected strains was evaluated as previously described by Guo et al. [20]. To measure acid tolerance, 1 mL of the strain suspension (adjusted to OD_600_ value of 1.0) was incubated with PBS (pH 2) at 37 °C for 3 h.

To measure bile salt tolerance, the strains were suspended in MRS broth containing 0–0.5% (w/v) bile salts (Oxgall) and incubated at 37 °C for 24 h. After incubation, the number of viable cells was counted. Cell viability was determined based on the cell counts on MRS agar plates and expressed as colony-forming units per mL (CFU/mL).

#### Hemolytic Activity

To determine hemolytic activity, the strains were streaked onto TSA medium containing 5% sheep blood and incubated at 37 °C for 48 h. The formation of a clear zone (β-hemolysis), a greenish zone (α-hemolysis), or no zone (γ-hemolysis) around the colonies was observed.

#### Auto-aggregation Assay

Auto-aggregation for the selected strains was conducted using the method described by Kos et al. [21]. Briefly, the strain cultures were inoculated (2%, v/v) into fresh MRS broth and incubated at 37 °C for 18 h. The suspension was centrifuged at 4000 × *g* for 15 min at 4 °C and washed twice with PBS. After resuspending the strains to a final concentration of OD_600_ 1.0, 4 mL of aliquots of the suspensions was shaken for 10 s, and auto-aggregation was measured for 5 h. Auto-aggregation (%) was calculated as follows:

5 $${\text{Auto - aggregation}}\,\left( \% \right)\, = \,\left[ {\frac{{(A\, - \,B)}}{A}} \right]\, \times \,100,$$

where *A* is the absorbance at 0 h of incubation and *B* is the absorbance after incubation for 1, 2, 3, 4, or 5 h.

### Antibiotic Susceptibility

The antibiotic susceptibility of the selected strains was evaluated using the minimum inhibitory concentration (MIC) test strip method. The susceptibility of the selected strains to the following 9 antimicrobial agents were tested: ampicillin, chloramphenicol, clindamycin, erythromycin, gentamicin, kanamycin, streptomycin, tetracycline, and vancomycin. The bacterial cells were incubated aerobically at 37 °C for 18 h in MRS medium. Next, cells were harvested by centrifugation at 3750 × *g* for 5 min, washed thrice with PBS, and resuspended in PBS to a McFarland standard of 0.5. The cell suspension was inoculated onto BHI agar with swabs. The plates were allowed to dry for 10 to 15 min, and the MIC test strips (Liofilchem, Italy) were placed on the agar surface. The plates were incubated at 37 °C, and MICs were analyzed after 48 h of incubation. MICs were determined based on the intersection of the elliptical zone of growth inhibition with the MIC scale on the test strip. The cut-off values for different antibiotics were evaluated according to European Food Safety Authority (EFSA) guidelines 2018 [22].

### Enzyme Production and Biochemical Profile Characterization

The enzyme activity and carbohydrate utilization of the selected strains were assayed using API ZYM and API 50CHL kit according to the manufacturer’s instructions (BioMérieux, France). Evaluation of enzyme activity was performed on a five-grade scale according to coloration intensity from 0 (no activity) to 5 (maximum activity) with 10 nM intervals. API strip reactions were evaluated using identification tables (+/−) according to color change.

### Statistical Analysis

Results are presented as the means ± standard deviation (SD) of experiments performed in triplicate. Graphical representation was generated using Prism software 9.0 (GraphPad Software, CA, USA). Statistical analysis was conducted using one-way analysis of variance (ANOVA) using SPSS (IBM Corp., USA). Significant differences between the groups were evaluated using Tukey’s multiple comparison test. Statistical significance was accepted for *P* values <0.05.

## Results

### α-Glucosidase and α-Amylase Inhibitory Activities of LAB Strains

The inhibitions of α-glucosidase and α-amylase delay glucose absorption and reduce postprandial blood glucose levels [23]. In this study, the α-glucosidase inhibitory activities of 235 strains were investigated to identify strains with potential hypoglycemic activity. Acarbose (an antidiabetic drug) and *Lacticaseibacillus (Lcb.) rhamnosus* GG (LGG), known to have an antidiabetic effect, were used as positive controls [24]. In total, 17 strains showed α-glucosidase inhibitory activity by more than 60% similar to those of acarbose (1000 μg/ mL) (Table 1). The four selected strains showed a high α-glucosidase inhibitory activity of more than 75%, which was approximately two times higher than that of LGG (36.7%).

**Table 1:** Inhibitory effects of the LAB strains against α-glucosidase and α-amylase

Origin	Strains	Inhibition (%)	
		α-glucosidase	α-amylase
Control	Acarbose (1,000 μg/mL)	50.9 ± 2.0	86.0 ± 0.6
	*Lcb. rhamnosus* GG	36.7 ± 7.3	86.3 ± 0.8
Human vagina	*Lpb. plantarum* MG4229	79.1 ± 6.0	85.6 ± 0.7
	*Lac. gasseri* MG4238	68.0 ± 2.5	84.7 ± 0.8
	*Lsb. fermentum* MG4290	64.8 ± 2.5	86.6 ± 0.5
	*Lsb. fermentum* MG4294	71.8 ± 4.8	88.7 ± 0.6
	*Lsb. fermentum* MG4295	70.6 ± 5.4	83.7 ± 1.5
	*Lpb. plantarum* MG4296	90.6 ± 1.6	86.2 ± 0.4
	*Lsb. fermentum* MG4302	67.2 ± 6.0	81.7 ± 0.4
	*Lpb. plantarum* MG4306	66.1 ± .4	57.0 ± 1.3
Infant	*Lcb. rhamnosus* MG4501	71.1 ± 6.2	62.6 ± 2.0
	*Lcb. rhamnosus* MG4502	63.4 ± 4.7	62.2 ± 0.7
Shellfish	*Lcb. paracasei* MG5004	70.7 ± 2.3	76.3 ± 0.6
	*Lcb. paracasei* MG5012	82.8 ± 3.4	87.4 ± 0.1
	*Lpb. plantarum* MG5025	77.3 ± 0.3	85.4 ± 0.2
Fermented food	*Lc. lactis* MG5127	64.7 ± 2.5	87.7 ± 0.2
	*Lpb. plantarum* MG5144	69.6 ± 3.1	83.2 ± 0.1
	*Lcb. paracasei* MG5172	62.5 ± 8.0	65.6 ± 2.8
	*Lcb. paracasei* MG5178	65.8 ± 3.8	79.7 ± 0.8

Results are presented as means ± SD from three independent experiments

*Lcb*. *Lacticaseibacillus*, *Lpb*. *Lactiplantibacillus*, *Lac*. *Lactobacillus*, *Lsb. Limosilactobacillus*, *Lc*. *Lactococcus*

Additionally, the α-amylase inhibitory activities of 17 strains ranged from 57.0 to 88.7%. MG4294 exhibited the highest α-amylase inhibitory activity, followed by MG5012. The α-amylase inhibitory activities of these two strains were similar to those of LGG (>85%) and acarbose (>86%).

### In Vitro Antioxidant Properties of the LAB Strains

The antioxidant capacity of the ICs of the eight selected strains exhibiting a high α-glucosidase inhibitory activity was compared with that of ascorbic acid (50 μg/mL) and LGG (Table 2). The highest DPPH radical scavenging activity was exhibited by MG4296 (75.8%). The DPPH radical scavenging activity of MG4229, MG4294, MG4501, and MG5004 were more than 60%. The ABTS radical scavenging activity of all eight strains was higher than that of ascorbic acid. MG4501 exhibited the highest ABTS radical scavenging activity. MG4229, MG4294, and MG5025 were also showed more than 50% activity. The highest FRAP value was exhibited by MG5012, which was similar to that of ascorbic acid.

**Table 2:** Antioxidant activities of the selected strains

Strains	DPPH radical scavenging (%)	ABTS radical Scavenging (%)	FRAP reducing power (μg/mL)
Ascorbic acid (50 μg/mL)	68.1 ± 2.7	30.1 ± 2.4	292.1 ± 4.4
*Lcb. rhamnosus* GG	44.3 ± 0.7	54.4 ± 1.8	263.7 ± 5.9
*Lpb. plantarum* MG4229	63.4 ± 0.9	53.4 ± 1.3	247.9 ± 6.4
*Lsb. fermentum* MG4294	66.7 ± 4.8	50.8 ± 0.9	279.9 ± 5.9
*Lsb. fermentum* MG4295	47.0 ± 1.9	46.8 ± 0.4	243.3 ± 10.1
*Lpb. plantarum* MG4296	75.8 ± 1.9	41.3 ± 1.5	220.0 ± 4.4
*Lcb. rhamnosus* MG4501	66.0 ± 1.3	60.4 ± 1.8	246.5 ± 9.5
*Lcb. paracasei* MG5004	60.8 ± 0.8	43.1 ± 0.9	241.7 ± 6.7
*Lcb. paracasei* MG5012	32.6 ± 6.1	47.1 ± 2.0	297.0 ± 3.3
*Lpb. plantarum* MG5025	25.0 ± 3.2	50.7 ± 1.1	247.4 ± 5.2

Results are presented as means ± SD from three independent experiments

*DPPH* 2,2-diphenyl-1-picrylhydrazyl, *ABTS* 2,2’-azino-bis(3-ethylbenzothiazoline-6-sulfonic acid), *FRAP* ferric reducing/antioxidant power, *Lcb*. *Lacticaseibacillus*, *Lpb*. *Lactiplantibacillus*, *Lsb. Limosilactobacillus*

### Identification of Selected LAB Strains

**Fig. 1:**
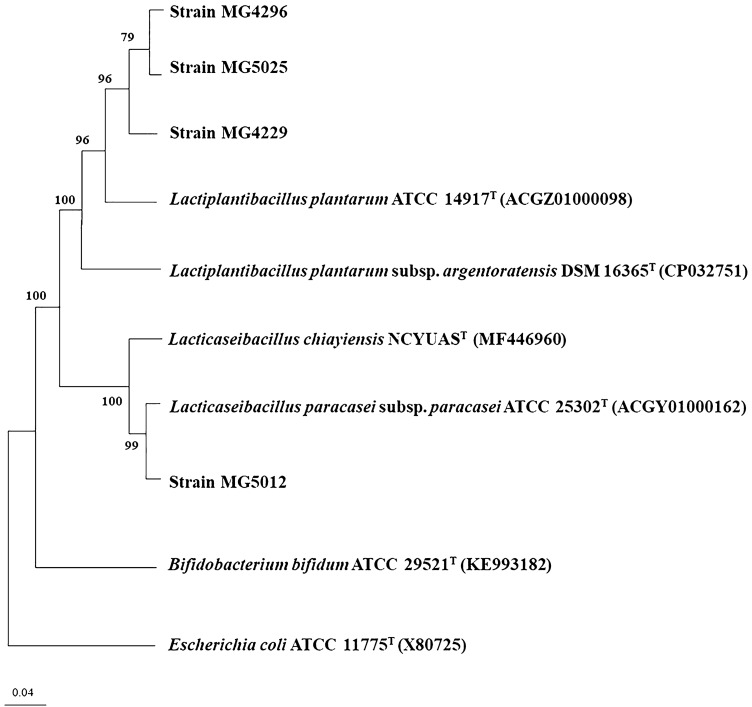
Phylogenetic tree constructed using the neighbor-joining, method with the 16S rRNA gene sequences. The correlation between the test strains (MG4229, MG4296, MG5025, and MG5012) and members of the genus *Lactiplantibacillus* and *Lacticaseibacillus*. Accession numbers are shown in parentheses. The sequence of *Bifidobacterium bifidum* ATCC 29521 T (KE993182) and *Escherichia coli* ATCC 11775 T (X80725) were used as outgroup. Bar, 0.04 nucleotide substitutions per site

Among the strains with potent biological activities, four strains (MG4229, MG4296, MG5012, and MG5025) were selected based on their α-glucosidase inhibitory and antioxidant activities. A phylogenetic tree constructed using 16S rRNA gene sequences revealed that the selected strains belonged to the cluster comprising *Lactobacillus plantarum* (recently reclassified as *Lactiplantibacillus plantarum)* and *Lactobacillus paracasei* (reclassified as *Lacticaseibacillus paracasei*) (Fig. 1) [25]. The GenBank accession numbers for the 16S rRNA gene sequences of the strains MG4229, MG4296, MG5012, and MG5025 are MN060991, MN060993, MN060994, and MN060995, respectively.

### Survival of the Selected Strains Under Simulated Gastrointestinal Conditions

Gastric tolerance of the selected four strains was expressed as the number of viable cells after exposure to simulated gastric juice (pH 2) for 0–3 h (Table 3). All strains exhibited similar viable cell counts, ranging from 5.0 to 5.7 log CFU/mL, under simulated gastric fluid conditions. The viability of all strains was higher than 57%.

**Table 3:** Tolerance of the selected strains to simulated gastric juice conditions

Strains	Exposure time (h)	Gastric tolerance (at pH 2)	
		Viable cells (log CFU/mL)	Survival rate (%)
*Lpb. plantarum* MG4229	0	8.6 ± 0.30	100
	1	6.0 ± 0.12	69.8
	2	5.2 ± 0.18	60.5
	3	5.3 ± 0.26	61.6
*Lpb. plantarum* MG4296	0	8.5 ± 0.14	100
	1	4.9 ± 0.13	57.6
	2	4.8 ± 0.02	56.5
	3	5.0 ± 0.24	58.8
*Lpb. plantarum* MG5025	0	8.2 ± 0.05	100
	1	6.1 ± 0.06	74.4
	2	5.8 ± 0.07	70.7
	3	5.7 ± 0.81	69.5
*Lcb. paracasei* MG5012	0	8.7 ± 0.06	100
	1	5.2 ± 0.04	59.8
	2	5.1 ± 0.11	58.6
	3	5.0 ± 0.26	57.5

Results are presented as means ± SD from three independent experiments. Simulated gastric tolerance was evaluated based on the viable cell counts (log CFU/mL) of each strain at pH 2 at 1 h intervals till 3 h

*Lcb*. *Lacticaseibacillus*, *Lpb*. *Lactiplantibacillus*

In addition, the cell viability of the selected strain was measured under bile salt stress conditions of 0–0.5% (Table 4). All strains exhibited a survival rate of more than 82% at a bile salt condition of 0.5%.

**Table 4:** Survival of the selected strains under bile salt stress conditions

Strains	Viable cells (log CFU/mL)				Survival rate at 0.5% bile salt (%)
	Bile salt (%)				
	0	0.1	0.3	0.5	
*Lpb. plantarum* MG4229	9.1 ± 0.11	8.6 ± 0.09	7.9 ± 0.10	7.8 ± 0.03	85.7
*Lpb. plantarum* MG4296	8.2 ± 0.26	7.0 ± 0.03	7.0 ± 0.20	7.0 ± 0.10	85.4
*Lpb. plantarum* MG5025	8.8 ± 0.15	8.2 ± 0.09	7.3 ± 0.11	7.2 ± 0.15	81.8
*Lcb. paracasei* MG5012	9.0 ± 0.21	9.0 ± 0.09	8.0 ± 0.13	7.4 ± 0.14	82.2

Results are presented as means ± SD from three independent experiments. Tolerance to simulated bile salt stress conditions was evaluated based on the viable cell counts (log CFU/mL) of each strain after 24 h of incubation at 37 °C

*Lcb*. *Lacticaseibacillus*, *Lpb*. *Lactiplantibacillus*

### Auto-aggregation of Selected Strains

**Fig. 2:**
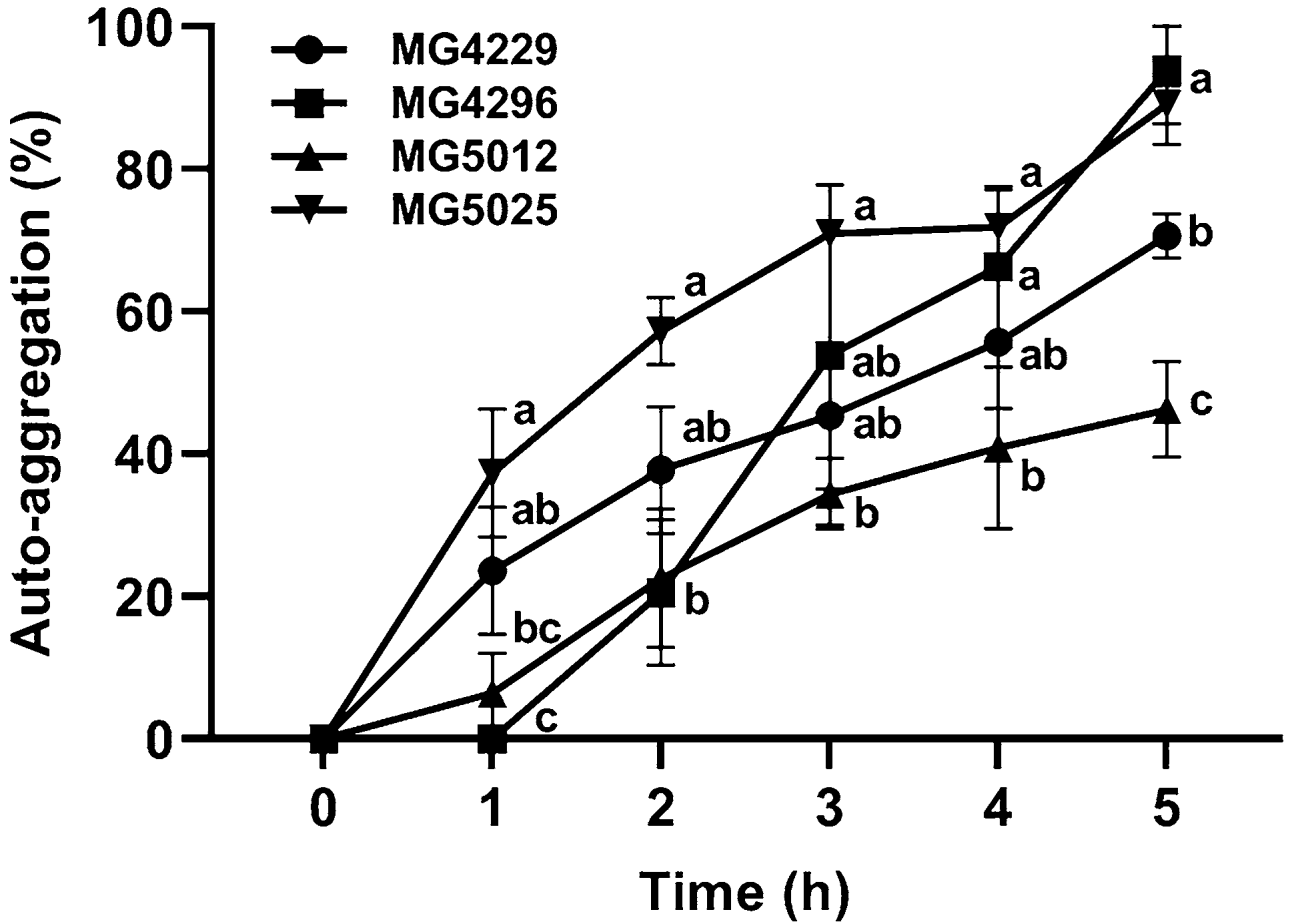
Auto-aggregation abilities of the selected strains resuspended in phosphate-buffered saline (pH 7) after 5 h. Results are presented as means ± SD from three independent experiments. Different letters at each time point indicate a significant difference (*P* <0.05) using Tukey’s multiple comparison test following a one-way ANOVA

To be classified as probiotics, LAB must reach the intestine through the stomach and duodenum and attach to the intestinal epithelial cells. In this study, the adherence ability of the four selected strains was tested by measuring auto-aggregation after 5 h of incubation (Fig. 2). The decreasing order of auto-aggregation of the strains was as follows: MG4296 (93.9 ± 10.5%) > MG5025 (89.1 ± 2.7%) > MG4229 (70.6 ± 3.1%) > MG5012 (46.2 ± 6.7%).

### Antibiotic Susceptibility of Selected Strains

Probiotics should be validated for their permeability, pathogenicity, and antibiotic resistance [26]. In this study, the antibiotic resistance of the selected strain was evaluated using the MIC test (Table S1). All strains were sensitive to most antibiotics and resistant to vancomycin. The MIC values were within the epidemiological cut-off values suggested by EFSA (2018). In addition to vancomycin, MG5012 was resistant to kanamycin.

### Carbohydrate Utilization and Enzyme Activity Profiles of Selected Strains

Carbohydrate utilization properties of the four strains were investigated using the API 50 CHL system (Table S2). All strains metabolized common carbohydrates, including d-ribose, d-galactose, d-glucose, d-fructose, and d-mannose. However, glycerol, erythritol, d-arabinose, d-xylose, l-xylose, methyl-β-d-xylopyranoside, l-rhamnose, dulcitol, inositol, starch, glycogen, xylitol, d-fucose, l-fucose, d-arabitol, l-arabitol, potassium 2-keto-gluconate, and potassium 5-keto-gluconate were not metabolized by these strains.

In addition, the enzyme activities of the four strains were assessed using the API ZYM system (Table S3). MG4296 and MG5025 exhibited the highest β-galactosidase and N-acetyl-β-glycosaminidase activities. MG4229 exhibited the highest leucine arylamidase activities. β-glucuronidase, a carcinogenic enzyme, hydrolyzes glucuronides into harmful carcinogenic substances, such as glucuronic acid and aglycone in the gut. The four strains did not produce β-glucuronidase, α-fucosidase, or α-mannosidase.

## Discussion

DM is characterized by hyperglycemia and insulin resistance, which result from dysregulated blood glucose homeostasis. The therapeutic strategies for T2DM include stimulating insulin secretion, increasing the activity of insulin in the target tissues, oral hypoglycemic agents, and inhibition of α-glucosidase [27]. Numerous studies have demonstrated that the α-glucosidase inhibitory and α-amylase inhibitory activities of probiotics may be beneficial for glycemic regulation [28, 29]. Probiotics have been reported to alleviate T2DM by regulating glucose metabolism and improving insulin sensitivity through several mechanisms, including the production of metabolites, such as SCFAs [30, 31]. Some probiotic strains have been reported to produce bioactive components capable of inhibiting α-glucosidase activity [15, 32]. Therefore, this study was aimed to identify functional probiotic candidate strains for improving diabetes by evaluating hypoglycemic and antioxidant activities. Additionally, the probiotic properties of the selected strains were evaluated.

α-glucosidase inhibitors reduce postprandial hyperglycemia by interfering with the activity of carbohydrate digestive enzymes and delaying the absorption of glucose [10]. Acarbose, an antidiabetic, is the most widely used α-glucosidase inhibitor to treat diabetes and acts by delaying the release of glucose from polysaccharides by biding to α-glucosidase [33]. In this study, α-glycosidase and α-amylase inhibitory activities of four selected strains (MG4229, MG4296, MG5012, and MG5025) were similar to those of acarbose (1000 μg/mL). In particular, MG4296 showed the highest inhibition on α-glycosidase (Table 1). In a previous study, Kim et al. [34] demonstrated that *Lactiplantibacillus (Lpb.) plantarum* K10 effectively inhibited α-amylase activity by 94.6%. Koh et al. [35] reported that the α-glucosidase inhibitory activities of culture supernatant of LGG and *Liquorilactobacillus mali* K8 were 37.9% and 39.0%, respectively. Ramchandran et al. [36] reported that exopolysaccharides produced by LAB strains inhibited α-glucosidase. These findings demonstrated that the selected strains exhibit hypoglycemic activity by inhibiting carbohydrate-metabolizing enzymes.

Oxidative stress is caused by an imbalance between reactive oxygen species (ROS) production and antioxidant defense mechanisms [37]. Previous studies have reported that oxidative stress induces DM complications [38, 39]. The antioxidant systems scavenge oxidizing compounds during the metabolic activity of probiotic bacteria or inhibit the production of oxidizing compounds in the intestine [40]. Biologically active peptides released from digested food proteins are reported to exhibit antioxidant activity and protect against peroxidation of lipids or fatty acids [41]. Some probiotics produce bioactive compounds that can reduce oxidative stress by preventing ROS formation through specific molecular mechanisms [42]. Similar to teichoic acid in the cell membrane and peptidoglycan in the cell wall, LAB exhibits antioxidant activity by chelating metal ions and exhibiting reducing activity [43]. *Lpb. plantarum* AS1 inhibited linoleic acid peroxidation by 50.9% [44]. *Lacticaseibacillus* (*Lcb.*) *paracasei* F19 alleviated oxidative and hepatic metabolic injuries in a rat model [45]. This study comparatively analyzed the free radical scavenging activities of ICs of selected eight strains, ascorbic acid, and LGG. The ABTS radical scavenging activity and FRAP values of these strains were similar to those of ascorbic acid (50 μg/mL). In a previous study, ICs of *Lactobacillus* (*Lac.*) *acidophilus* and *Lcb. paracasei* showed the DPPH radical scavenging activities range of 31–48% [8]. *Limosilactobacillus reuteri* and *Bifidobacterium breve* are reported to exhibit ABTS radical scavenging activities. Afify et al. [46] reported that *Lpb. plantarum* C88 showed high hydroxyl radical (44.3%) and DPPH scavenging (53.1%) activities. Therefore, these findings demonstrate that the selected strains exhibit potential antioxidant activity.

Probiotics should survive in the human gastrointestinal environment. Ingested LAB is exposed to gastric fluid, bile, and digestive enzymes and subsequently attaches to the intestine [47]. Thus, the function of the ingested probiotics is dependent on their growth in an environment with higher bile concentrations and lower pH (pH of gastric juice is <3) than those in the intestine [48]. In this study, the viable counts of selected strains were more than 5.0 log CFU/mL in simulated gastric fluid. MG5025 exhibited the highest viability, approximately 70%. The high acid resistance of lactobacilli strains has been reported to be associated with changes in glycolytic flux, intracellular pH, and cell membrane ATPase [49]. The viable count of several strains used as probiotics was less than 10^4^ CFU/mL at pH 2 [50]. Therefore, these results suggest that the selected strains are resistant to acidic conditions.

Probiotics are exposed to various stress conditions, including heat, oxidative, osmotic, and bile salt stresses [51]. Generally, microbes that can survive at bile salt concentrations of up to 0.3% are reported to exhibit bile salt tolerance [52]. Various species of LAB exhibit bile salt tolerance as they produce bile salt hydrolase, which hydrolyzes bile acids [53]. In this study, all selected strains showed a high number of viable cells at bile salt concentrations of 0.3% (>8.0 log CFU/mL) and 0.5% bile salt (>6.86 log CFU/mL) in an artificial intestinal environment. Therefore, these findings suggest that the selected strains can survive in the human intestinal environment.

Auto-aggregation is a prerequisite for colony formation and probiotic strain persistence in the gastrointestinal system. LAB is attached to specific and non-specific tissues in the gut. Proteins, glycoproteins, teichoic acid, and lipoteichoic acid on bacterial cell wall surfaces are known to play important roles in auto-aggregation and hydrophobicity [54]. In this study, the auto-aggregation rates of MG4296 and MG5025 were as high as 90% (Fig. 2). In a previous study, Al et al. [55] reported that the auto-aggregation rate of human-derived *Lac. acidophilus* CMUL67 was 76.2%. García-Cayuela et al. [56] reported the auto-aggregation rate of four species of *Lpb. plantarum* was more than 50%. Therefore, the strains selected in this study can adhere to the intestinal epithelial cells and exhibited a high survival rate in the simulated intestinal environment.

Probiotics are inherently resistant to various antibiotics. The survival, proliferation, and functions of the bacterial cells are dependent on their resistance to antibiotics [57]. In this study, the four selected strains of *Lpb. plantarum* and *Lcb. paracasei* were resistant to vancomycin. Previous studies have demonstrated that *Lpb. plantarum*, *Lcb. paracasei*, *Lcb. rhamnosus,* and *Lac. acidophilus* are resistant to vancomycin.

Microorganisms produce and inhibit enzyme activity through unique mechanisms [58]. In this study, MG4229, MG4296, and MG5025 were found to contain more than 40 nM β-galactosidase (Table 4). The production of some enzymes in probiotics should be evaluated to prevent the synthesis of potentially toxic substances or harmful metabolites, such as indole and amines [59]. β-glucuronidase, a carcinogenic enzyme, hydrolyzes glucuronides into harmful carcinogenic substances, such as glucuronic acid and aglycone in the gut [60]. In this study, the selected strains did not produce β-glucuronidase. Therefore, these findings indicated the safety of the selected strain.

## Conclusion

The present study was conducted to select the probiotic strains for industrial applicability as a functional agent with antidiabetic and antioxidant activities. We identified four strains (*Lpb.* *plantarum* MG4229, MG4296, MG5025, and* Lcb. paracasei* MG5012) exhibiting potent α-glucosidase inhibitory, α-amylase inhibitory, and antioxidant activities. The probiotic properties and safety of these strains were also demonstrated. Further studies are needed to determine the efficacy of these strains in an in vivo T2DM model. However, these results of this study demonstrated that the selected strains were potential probiotic candidates with antidiabetic activity.

## Supplementary Information

Below is the link to the electronic supplementary material.

Supplementary file1 (DOCX 23 KB)

## References

[1] Wegh CA, Geerlings SY, Knol J, Roeselers G, Belzer C (2019). Postbiotics and their potential applications in early life nutrition and beyond. Int J Mol Sci.

[2] Masood MI, Qadir MI, Shirazi JH, Khan IU (2011). Beneficial effects of lactic acid bacteria on human beings. Crit Rev Microbiol.

[3] Jandhyala SM, Talukdar R, Subramanyam C, Vuyyuru H, Sasikala M, Nageshwar Reddy D (2015). Role of the normal gut microbiota. World J Gastroenterol.

[4] Punthakee Z, Goldenberg R, Katz P (2018). Definition, classification and diagnosis of diabetes, prediabetes and metabolic syndrome. Can J Diabetes.

[5] Gurung M, Li Z, You H, Rodrigues R, Jump DB, Morgun A, Shulzhenko N (2020). Role of gut microbiota in type 2 diabetes pathophysiology. EBioMedicine.

[6] Tabrizi R, Moosazadeh M, Lankarani KB, Akbari M, Heydari ST, Kolahdooz F, Asemi Z (2018). The effects of synbiotic supplementation on glucose metabolism and lipid profiles in patients with diabetes: a systematic review and meta-analysis of randomized controlled trials. Probiotics Antimicro.

[7] Gérard C, Vidal H (2019). Impact of gut microbiota on host glycemic control. Front Endocrinol.

[8] Lin MZ, Chai WM, Zheng YL, Huang Q, Ou-Yang C (2019). Inhibitory kinetics and mechanism of rifampicin on α-glucosidase: Insights from spectroscopic and molecular docking analyses. Int J Biol Macromol.

[9] Choi K, Choi SI, Park MW, Han JS (2017). Cyanidin-3-O-glucoside ameliorates postprandial hyperglycemia in diabetic mice. J Life Sci.

[10] Krentz AJ, Bailey CJ (2005). Oral antidiabetic agents: current role in Type 2 diabetes melitus. Drugs.

[11] Kumar S, Narwal S, Kumar V, Prakash O (2011). α-glucosidase inhibitors from plants: a natural approach to treat diabetes. Pharmacogn Rev.

[12] Dileep KV, Nithiyanandan K, Remya C (2018). Binding of acarbose, an anti-diabetic drug to lysozyme: a combined structural and thermodynamic study. J Biomol Struct Dyn.

[13] Tamura K, Peterson D, Peterson N, Stecher G, Nei M, Kumar S (2011). MEGA5: molecular evolutionary genetics analysis using maximum likelihood, evolutionary distance, and maximum parsimony methods. Mol Biol Evol.

[14] Lin MY, Chang FJ (2000). Antioxidative effect of intestinal bacteria *Bifidobacterium longum* ATCC 15708 and *Lactobacillus acidophilus* ATCC 4356. Digest Dis Sci.

[15] Chen P, Zhang Q, Dang H, Liu X, Tian F, Zhao J, Chen Y, Zhang H, Chen W (2014). Screening for potential new probiotic based on probiotic properties and α-glucosidase inhibitory activity. Food Control.

[16] Vankudre M, Balpande A, Athale M (2015). Comparative analysis of α-amylase inhibition and antioxidant activity of whey from cow and buffalo milk fermented with *lactobacillus* species. Biosci Biotechnol Res Comm.

[17] Lim SM (2010). Resistance to reactive oxygen species and antioxidant activities of some strains of lactic acid bacteria from the mustard leaf kimchi. Korean J Microbiol.

[18] Re R, Pellegrini N, Proteggente A, Pannala A, Yang M, Rice-Evans C (1999). Antioxidant activity applying an improved ABTS radical cation decolorization assay. Free Radical Bio Med.

[19] Benzie IF, Strain JJ (1996). The ferric reducing ability of plasma (FRAP) as a measure of antioxidant power: the FRAP assay. Anal Biochem.

[20] Guo XH, Kim JM, Nam HM, Park SY, Kim JM (2010). Screening lactic acid bacteria from swine origins for multistrain probiotics based on in vitro functional properties. Anaerobe.

[21] Kos B, Šušković J, Vuković S, Šimpraga M, Frece J, Matošić S (2003). Adhesion and aggregation ability of probiotic strain *Lactobacillus acidophilus* M92. J Appl Microbiol.

[22] EFSA (2018). Guidance on the characterisation of microorganisms used as feed additives or as production organisms. EFSA J.

[23] Gazza L, Gazzelloni G, Taddei F, Latini A, Muccilli V, Alfieri M, Conti S, Redaelli R, Pogna NE (2016). The starch-bound alpha-amylase/trypsin-inhibitors in Avena. Mol Genet Genom.

[24] Kim SW, Park KY, Kim B, Kim E, Hyun CK (2013). *Lactobacillus rhamnosus* GG improves insulin sensitivity and reduces adiposity in high-fat diet-fed mice through enhancement of adiponectin production. Biochem Biophys Res Commun.

[25] Zheng J, Wittouck S, Slavetti E, Franz CMAP, Harris HMB, Mattarelli P, O’Toole PW, Pot B, Vandamme P, Walter J, Watanabe K, Wuyts S, Felis GE, Gänzle MG, Lebeer S (2020). A taxonomic note on the genus *Lactobacillus*: description of 23 novel genera, emended description of the genus *Lactobacillus* Beijerinck 1901, and union of *Lactobacillaceae* and *Leuconostocaceae*. Int J Syst Evol Microbiol.

[26] Gueimonde M, Sánchez B, de los Reyes-Gavilán CG, Margolles A (2013). Antibiotic resistance in probiotic bacteria. Front Microbiol.

[27] Nurhayati R, Frediansyah A, Rachmah DL (2017). Lactic acid bacteria producing inhibitor of alpha glucosidase isolated from Ganyong (*Canna Edulis*) and Kimpul (*Xanthosoma sagittifolium*). IOP Conf Ser Earth Environ Sci.

[28] Gazza L, Gazzelloni G, Taddei F, Latini A, Muccilli V, Alfieri M, Conti S, Redaelli R, Pogna NE (2016). The starch-bound alpha-amylase/trypsin-inhibitors in Avena. Mol Genet Genom Med.

[29] Panwar H, Calderwood D, Grant IR, Grover S, Green BD (2014). *Lactobacillus* strains isolated from infant feces possess potent inhibitory activity against intestinal alpha- and beta-glucosidases suggesting anti-diabetic potential. Eur J Nutr.

[30] Morrison DJ, Preston T (2016). Formation of short chain fatty acids by the gut microbiota and their impact on human metabolism. Gut Microbes.

[31] Herrema H, Niess JH (2020). Intestinal microbial metabolites in humna metabolism and type 2 diabetes. Dieteologia.

[32] Zeng Z, Luo J, Zuo F, Zhang Y, Ma H (2016). Screening for potential novel probiotic *Lactobacillus* strains based on high dipeptidyl peptidase IV and α-glucosidase inhibitory. J Funct Foods.

[33] Ghani U (2015). Re-exploring promising α-glucosidase inhibitors for potential development into oral anti-diabetic drugs: finding needle in the haystack. Eur J Med Chem.

[34] Kim S, Huang E, Park S, Holzapfel W, Lim SD (2018). Physiological characteristics and anti-obesity effect of *Lactobacillus plantarum* K10. Korean J Food Sci Anim Resour.

[35] Koh WY, Utra U, Ahmad R, Rather IA, Park YH (2018). Evaluation of probiotic potential and anti-hyperglycemic properties of a novel *Lactobacillus* strain isolated from water kefir grains. Food Sci Biotechnol.

[36] Ramchandran L, Shah NP (2009). Effect of exopolysaccharides and inulin on the proteolytic, angiotensin-I-converting enzyme-and α-glucosidase-inhibitory activities as well as on textural and rheological properties of low-fat yogurt during refrigerated storage. Dairy Sci Technol.

[37] Kumar V, Khan AA, Tripathi A, Dixit PK, Bajaj UK (2015). Role of oxidative stress in various diseases: relevance of dietary antioxidants. J Phytopharm.

[38] Bandeira DM, Da Fonseca LJS, Guedes DS, Rabelo LA, Goulart MO, Vasconcelos SML (2013). Oxidative stress as an underlying contributor in the development of chronic complications in diabetes mellitus. Int J Mol Sci.

[39] Luc K, Schramm-Luc A, Guzik TJ, Mikolajczyk TP (2019). Oxidative stress and inflammatory markers in prediabetes and diabetes. J Physiol Pharmacol.

[40] Azcárate-Peril MA, Sikes M, Bruno-Bárcena JM (2011). The intestinal microbiota, gastrointestinal environment and colorectal cancer: a putative role for probiotics in prevention of colorectal cancer?. Am J Physiol Gastrointest Liver Physiol.

[41] Virtanen T, Pihlanto A, Akkanen S, Korhonen H (2007). Development of antioxidant activity in milk whey during fermentation with lactic acid bacteria. J Appl Microbiol.

[42] Wang Y, Wu Y, Wang Y, Xu H, Mei X, Yu D, Wang Y, Li W (2017). Antioxidant properties of probiotic bacteria. Nutrients.

[43] Thomas KJ, Rice CV (2015). Equilibrium binding behavior of magnesium to wall teichoic acid. Biochim Biophys Acta Biomembr.

[44] Kumar RS, Kanmani P, Yuvaraj N, Paari KA, Pattukumar V, Thirunavukkarasu C, Arul V (2012). *Lactobacillus plantarum* AS1 Isolated from South Indian fermented food Kallappams suppress 1,2-dimethyl hydrazine (DMH)-induced colorectal cancer in male Wistar rats. Appl Biochem Biotechnol.

[45] Kumar M, Kumar A, Nagpal R, Mohania D, Behare P, Verma V, Kumar P, Poddar D, Aggarwal PK, Henry CJ, Jain S, Yadav H (2010). Cancer-preventing attributes of probiotics: an update. Int J Food Sci Nutr.

[46] Afify AEMR, Romeilah RM, Sultan SIM, Hussein MM (2012). Antioxidant activity and biological evaluations of probiotic bacteria strains. Int J Acad Res.

[47] Sim JH, Oh SJ, Kim SK, Baek YJ (1995). Comparative tests on the acid tolerance of some lactic-acid-bacteria species isolated from lactic fermented products. Korean J Food Sci Technol.

[48] Bang JH, Shin HJ, Choi HJ, Kim DW, Ahn CS, Jeong YK, Joo WH (2012). Probiotic potential of *Lactobacillus* isolates. J Life Sci.

[49] Radulović Z, Miočinović J, Mirković N, Mirković M, Paunović D, Ivanović M, Seratlić S (2017). Survival of spray-dried and free-cells of potential probiotic *Lactobacillus plantarum* 564 in soft goat cheese. Anim Sci J.

[50] Mishra V, Prasad DN (2005). Application of *in vitro* methods for selection of *Lactobacillus casei* strains as potential probiotics. Int J Food Microbiol.

[51] Kim SW, Perl L, Park JH, Tandianus JE, Dunn NW (2001). Assessment of stress response of the probiotic *Lactobacillus acidophilus*. Curr Microbiol.

[52] Lee YR, Son YJ, Park SY, Jang EY, Yoo JY, Son HJ (2016). Probiotic potential of plant-derived lactic acid bacteria with antihypertensive activity. Int J Environ Sci.

[53] Sahadeva RPK, Leong SF, Chua KH, Tan CH, Chan HY, Tong EV, Wong SYW, Chan HK (2011). Survival of commercial probiotic strains to pH and bile. Int Food Res J.

[54] Ferreira CL, Grześkowiak Ł, Collado MC, Salminen S (2011). In vitro evaluation of *Lactobacillus gasseri* strains of infant origin on adhesion and aggregation of specific pathogens. J Food Prot.

[55] Al Kassaa I, Hamze M, Hober D, Chihib NE, Drider D (2014). Identification of vaginal *lactobacilli* with potential probiotic properties isolated from women in North Lebanon. Microb Ecol.

[56] García-Cayuela T, Korany A, Martínez-Cuesta MC (2014). Adhesion abilities of dairy *Lactobacillus plantarum* strains showing an aggregation phenotype. Food Res Int.

[57] EFSA (2012). Guidance on the assessment of bacterial susceptibility to antimicrobials of human and veterinary importance. EFSA J.

[58] Seo JW, Yang HJ, Jeong SJ, Ryu MS, Ha G, Jeong SY, Jeong DY (2018). Characterization of *Lactobacillus brevis* SCML 432 isolated from Meju in Sunchang and optimization of its culture conditions by statistical methods. Korean J Food Preserv.

[59] Pessione E, Cirrincione S (2016). Bioactive molecules released in food by lactic acid bacteria: encrypted peptides and biogenic amines. Front Microbiol.

[60] Mroczynska M, Libudzisz Z (2010). Beta-glucuronidase and beta-glucosidase activity of *Lactobacillus* and *Enterococcus* isolated from human feces. Pol J Microbiol.

