# Supplementary material for: In Vitro Antidiabetic, Antioxidant Activity, and Probiotic Activities of Lactiplantibacillus plantarum and Lacticaseibacillus paracasei Strains
Source: Curr Microbiol. 2021 Jul 2;78(8):3181–91. doi: 10.1007/s00284-021-02588-5 (PMC8289794; doi:10.1007/s00284-021-02588-5)
Supplement: Supplementary file 1 — Supplementary file1 (DOCX 23 KB) [file 284_2021_2588_MOESM1_ESM.docx]

**Supplementary materials**

**Table S1** Minimum inhibitory concentration (MIC) of antibiotics for the selected strains

| **Antibiotics** | ***Lpb. plantarum***  **MG4229** | ***Lpb. plantarum***  **MG4296** | ***Lpb. plantarum***  **MG5025** | ***Lcb. paracasei***  **MG5012** |
| --- | --- | --- | --- | --- |
| Ampicillin | S | S | S | S |
| Chloramphenicol | S | S | S | S |
| Clindamycin | S | S | S | S |
| Erythromycin | S | S | S | S |
| Gentamicin | S | S | S | S |
| Kanamycin | S | S | S | R |
| Streptomycin | S | S | S | S |
| Tetracycline | S | S | S | S |
| Vancomycin | R | R | R | R |

Antibiotic susceptibilities were determined based on the guidelines of the EFSA (2018). The inhibitory zone was measured and determined according to the standard index. S, susceptibility, R, resistant. *Lpb*., *Lactiplantibacillus*; *Lcb*., *Lacticaseibacillus*.

**Table S2** Carbohydrate utilization profiles of the selected strains obtained using the API 50 CHL system

| **Carbohydrates** | ***Lpb. plantarum***  **MG4229** | ***Lpb. plantarum***  **MG4296** | ***Lpb. plantarum***  **MG5025** | ***Lcb. paracasei***  **MG5012** |
| --- | --- | --- | --- | --- |
| L-arabinose | + | − | − | − |
| D-ribose | + | + | + | + |
| D-adonitol | − | − | − | + |
| D-galactose | + | + | + | + |
| D-glucose | + | + | + | + |
| D-fructose | + | + | + | + |
| D-mannose | + | + | + | + |
| L-sorbose | − | − | − | + |
| D-mannitol | + | + | + | + |
| D-sorbitol | + | + | + | + |
| Methyl-α-D-mannopyranoside | + | + | + | − |
| Methyl-α-D-glucopyranoside | − | − | − | + |
| N-Acetyl-glucosamine | + | + | + | + |
| Amygdaline | + | + | + | + |
| Arbutin | + | + | + | + |
| Esculin | + | + | + | + |
| Salicin | + | + | + | + |
| D-cellobiose | + | + | + | + |
| D-maltose | + | + | + | + |
| D-lactose | + | + | + | − |
| D-melibiose | + | + | + | − |
| D-saccharose | + | + | + | + |
| D-trehalose | + | + | + | + |
| Inulin | − | − | − | + |
| D-melezitose | + | − | + | + |
| D-raffinose | + | + | + | − |
| Gentiobiose | + | + | + | + |
| D-turanose | + | − | − | + |
| D-lyxose | − | − | − | + |
| D-tagatose | − | − | − | + |
| Potassium gluconate | + | + | + | − |

Carbohydrate utilization of the strains was assayed using an API 50 CHL kit. +: Positive, the ability of the strains to fermented substrate, and −: Negative, inability of the strains to ferment substrate. All strains did not ferment Gycerol, Erythritol, L-xylose, Methyl-β-D-xylopyranoside, L-rhamnose, Dulcitol, Inositol, D-fucose, L-fucose, D-arabitol, L-arabitol, Starch, Glycogen, and 5-keto-gluconate. *Lpb*., *Lactiplantibacillus*; *Lcb*., *Lacticaseibacillus*.

**Table S3** Enzymatic activities of the selected strains assayed by using the API ZYM system

| **Enzyme** | ***Lpb. plantarum***  **MG4229** | ***Lpb. plantarum***  **MG4296** | ***Lpb. plantarum***  **MG5025** | ***Lcb. paracasei***  **MG5012** |
| --- | --- | --- | --- | --- |
| Control | 0 | 0 | 0 | 0 |
| Alkaline phosphatase | 1 | 0 | 1 | 1 |
| Esterase (C4) | 1 | 0 | 1 | 3 |
| Esterase Lipase (C8) | 1 | 0 | 1 | 3 |
| Lipase (C14) | 1 | 0 | 1 | 0 |
| Leucine arylamidase | 5 | 3 | 4 | 3 |
| Valine arylamidase | 4 | 0 | 1 | 4 |
| Crystine arylamidase | 1 | 0 | 1 | 2 |
| Trypsin | 1 | 0 | 0 | 1 |
| α-chymotrypsin | 1 | 0 | 0 | 2 |
| Acid phosphatase | 3 | 3 | 3 | 3 |
| Naphthol-AS-B1-hosphohydrolase | 2 | 2 | 0 | 3 |
| α-galactosidase | 1 | 2 | 3 | 1 |
| β-galactosidase | 5 | 5 | 5 | 2 |
| β-glucuronidase | 0 | 0 | 0 | 0 |
| α-glucosidase | 3 | 0 | 4 | 4 |
| β-glucosidase | 4 | 4 | 4 | 3 |
| N-acetyl-β-glucosaminidase | 3 | 5 | 5 | 0 |
| α-mannosidase | 0 | 0 | 0 | 0 |
| α-fucosidase | 0 | 0 | 0 | 0 |

The enzyme activities were recorded from 0 (no activity) to 5 (≥ 40 nM of product released) at 10 nM intervals in the API-ZYM color reaction chart. 0: No activity, 1: 5 nM, 2: 10 nM, 3: 20 nM, 4: 30 nM, and 5: ≥ 40 nM. Control: No Substrate. *Lpb*., *Lactiplantibacillus*; *Lcb*., *Lacticaseibacillus*.
